# Barriers of attendance to dog rabies static point vaccination clinics in Blantyre, Malawi

**DOI:** 10.1371/journal.pntd.0006159

**Published:** 2018-01-11

**Authors:** Stella Mazeri, Andrew D. Gibson, Natascha Meunier, Barend M.deC Bronsvoort, Ian G. Handel, Richard J. Mellanby, Luke Gamble

**Affiliations:** 1 The Roslin Institute, Division of Genetics and Genomics, Easter Bush Veterinary Centre, Roslin, Midlothian, United Kingdom; 2 The Royal (Dick) School of Veterinary Studies, The University of Edinburgh, Easter Bush Veterinary Centre, Roslin, Midlothian, United Kingdom; 3 Mission Rabies, Cranborne, Dorset, United Kingdom; 4 The Royal (Dick) School of Veterinary Studies, Division of Veterinary Clinical Studies, The University of Edinburgh, Hospital for Small Animals, Easter Bush Veterinary Centre, Roslin, Midlothian, United Kingdom; University of California San Francisco, UNITED STATES

## Abstract

Rabies is a devastating yet preventable disease that causes around 59,000 human deaths annually. Almost all human rabies cases are caused by bites from rabies-infected dogs. A large proportion of these cases occur in Sub Saharan Africa (SSA). Annual vaccination of at least 70% of the dog population is recommended by the World Health Organisation in order to eliminate rabies. However, achieving such high vaccination coverage has proven challenging, especially in low resource settings. Despite being logistically and economically more feasible than door-to-door approaches, static point (SP) vaccination campaigns often suffer from low attendance and therefore result in low vaccination coverage. Here, we investigated the barriers to attendance at SP offering free rabies vaccinations for dogs in Blantyre, Malawi. We analysed data for 22,924 dogs from a city-wide vaccination campaign in combination with GIS and household questionnaire data using multivariable logistic regression and distance estimation techniques. We found that distance plays a crucial role in SP attendance (i.e. for every km closer the odds of attending a SP point are 3.3 times higher) and that very few people are willing to travel more than 1.5 km to bring their dog for vaccination. Additionally, we found that dogs from areas with higher proportions of people living in poverty are more likely to be presented for vaccination (ORs 1.58-2.22). Furthermore, puppies (OR 0.26), pregnant or lactating female dogs (OR 0.60) are less likely to be presented for vaccination. Owners also reported that they did not attend an SP because they were not aware of the campaign (27%) or they could not handle their dog (19%). Our findings will inform the design of future rabies vaccination programmes in SSA which may lead to improved vaccination coverage achieved by SP alone.

## Author summary

Rabies is a devastating yet preventable disease that causes around 59,000 human deaths annually of which a large proportion occurs in Sub Saharan Africa (SSA). In order to eliminate rabies, annual vaccination of at least 70% of the dog population is recommended. In SSA most rabies vaccination programmes use static point (SP) vaccination approaches. Despite being logistically and economically more feasible than door-to-door approaches, SP vaccination campaigns often result in low vaccination coverage. Here we investigated the reasons why attendance at SPs offering free rabies vaccinations for dogs is suboptimal in SSA. We analysed data from a citywide vaccination campaign in Blantyre city, Malawi in combination with household related data. Our results found that the distance from home to SP influences attendance at SPs. We also found a clear need for provision of timely and accurate information about upcoming campaigns, including information on the importance of puppies being vaccinated as well as ways to improve dog handling. Understanding the barriers to attendance at SPs and taking them into consideration, would make mass vaccination programmes more feasible thereby allowing high vaccination coverage to be achieved without the need for expensive and logistically challenging door-to-door programmes.

## Introduction

Rabies has been estimated to cause around 59,000 human deaths per year [1]. Globally, rabies has been estimated to cause 3.7 million disability-adjusted life years and 8.6 billion US dollars economic losses annually [1]. Almost all human rabies cases are acquired from contact with rabies infected dogs [2]. Case fatality for patients who develop clinical signs related to rabies infection approaches 100% and successful treatment has rarely been reported [2]. Rabies disproportionately affects Sub Saharan African countries [1, 2]. Despite significant regional and international healthcare intervention initiatives, no African country has been reported rabies free to date [3].

Since 99% of all human rabies deaths are caused by bites from rabies infected dogs [2], mass dog vaccination campaigns are the single most effective strategy to eliminate rabies amongst humans and dogs [1, 4, 5]. To effectively eliminate rabies from canine and human populations, a critical requirement of mass dog vaccination programmes is to ensure that a sufficiently high proportion of dogs are vaccinated [6]. Empirical data has shown that annual vaccination coverage of 70% is sufficient to eliminate rabies from dog and human populations [6, 7]. This has been further validated by mathematical modelling [8]. For example, mathematical models have demonstrated that a cut-off of 70% would prevent a major disease outbreak at least 96.5% of the time based on rabies field data from USA, Mexico, Malaysia and Indonesia [8]. Collectively, these findings have resulted in the recommendation by the World Health Organisation (WHO) that rabies vaccination programmes should vaccinate at least 70% of all dogs annually [6, 7, 9, 10].

However, vaccinating large numbers of dogs at over 70% coverage has proved challenging despite the development of a range of mass rabies vaccination strategies [11]. Vaccination approaches which have been used include door-to-door campaigns (D2D); static point (SP) campaigns, using both fixed and temporary posts; and a combination of the two. Door-to-door programmes, which typically achieve a high vaccination coverage, are labour intensive, expensive and challenging to roll out on a large scale. Consequently, most rabies vaccination programmes in Sub Saharan Africa (SSA) have used SP vaccination approaches where the vaccination teams remain at a static location within a community and the local inhabitants present dogs to the vaccination teams. Although widely used in Africa as they are logistically and economically more feasible than door-to-door approaches, SP approaches have often failed to reach a high coverage [9, 11–14]. Consequently, many organisations have to manage a trade off when rolling out dog vaccination programmes of either utilising a door-to-door approach, which typically results in high coverage but lower numbers of dogs vaccinated, or a SP approach, which often achieves a lower coverage but facilitates the vaccination of more dogs.

This trade-off between coverage and number of dogs vaccinated would be eliminated if a higher proportion of dogs could be vaccinated through SPs. The reasons why attendance at SPs is low in many countries has surprisingly received little attention despite it being a major reason why rabies elimination programmes have been so challenging to effectively roll out in SSA. If the barriers to attendance at SPs could be understood and then overcome, mass vaccination programmes could become more feasible thereby allowing high vaccination coverage to be achieved without the need for expensive and logistically challenging door-to-door programmes.

Only a small number of studies have explored why attendance to SPs is often suboptimal. In previous small scale studies in Chad, Mali, Peru and urban Tanzania the most common reasons reported by dog owners for not attending a static vaccination point included; lack of information about the campaign [14–18], difficulty in handling dogs [13–15, 17, 18], lack of time [14, 17, 18], lack of information about rabies [15], mistrust [15], distance/location of SP [13, 15, 18], the dog being too young [16–18] or lactating [17, 18] and the lack of money to pay [14, 16]. In campaigns using a combination of SPs and D2D vaccination strategies, it is also possible that owners do not attend SPs as they expect to get their dogs vaccinated during the door-to-door campaign [16]. Furthermore, while giving out dog collars or wristbands can increase participation [18], charging the owners for vaccinations can result in lower vaccination rates [16]. Despite these studies, there is still an incomplete understanding of the barriers which limit attendance at static points.

The need to understand and overcome barriers to SP attendance is particularly important in Blantyre, Malawi where rabies is an important cause of mortality, especially in children [19]. In order to address the high incidence of rabies in this population, we have embarked on an annual mass dog vaccination campaign throughout the city. We have previously reported that although 97% of the dog population is owned, only 53% out of the 79% overall vaccination coverage we achieved in our 2015 vaccination programme was attributed to dogs vaccinated at a SP, with the remaining 26% achieved by vaccinating dogs at door-to-door [20].

In order to make the vaccination campaign financially sustainable in the longer term, we need to reduce the reliance on D2D vaccination and encourage higher attendance at SP vaccination stations. Consequently, the aim of this study was to investigate the barriers to attendance at SP vaccination clinics using a multi-faceted approach including modeling the relationship between distance to travel and attendance at SP together with dog owner questionnaires. Our study is the first large scale, city-wide study investigating the reasons for failure to attend static vaccination points in SSA.

## Materials and methods

### Ethics statement

Prior to vaccination of owned dogs, verbal informed consent was obtained from the person presenting the dog for vaccination. In the cases where an owner could not be identified, dogs were vaccinated in accordance with Government Public Health protocol, as the work was part of a non-research public health campaign.

### Study site

This study was conducted in Blantyre city, the second largest city in Malawi with an estimated human population of 881,074 in 2015 [21]. The city’s dog population in 2015 was estimated to be 45,526 based on mark re-sight methods [20]. The city covers an area of 220 km^2^, which is divided into 25 administrative wards [22]. The campaign took place throughout the whole of Blantyre city.

### Vaccination campaign—SP and D2D

The vaccination campaign has been described in detail by Gibson et al. [20]. Briefly, the city was divided in 204 working zones and their sizes were subjectively dictated according to an area that could be covered by a vaccination team in one day. Each zone was assigned a land type based on appearance in Google Satellite Maps^™^: a) housing category (HS) 1 (small houses—high density), b) HS 2 (small houses—medium density), c) HS 3 (small houses-low density), d) HS 4 (medium houses—ordered), e) HS 5 (large houses-medium/low density), f) industrial/commercial, g) agriculture/open space. For the purposes of the regression analysis described below these were regrouped in high (a), medium (b,d) and low (c,e,f,g) housing density areas.

Mass dog vaccination across the city was carried out between the 30th of April and the 25th of May 2016 using two approaches; static point (SP) and door-to-door (D2D). Using 8 vaccination teams working simultaneously, SP vaccinations were conducted at weekends followed by D2D vaccinations in the same area on the following Monday, Tuesday and Wednesday.

### Data sources

#### Data collection

During the D2D campaign data were collected on every dog encountered using the Mission Rabies (MR) Smartphone App [20]. Relevant data for this study include the age, sex, pregnancy and lactation status of the dog, confinement level, GPS location, whether the dog has been vaccinated at a SP or by someone other than MR, and reasons for not attending SP clinics. For a subset of these dogs, information on which specific SP they attended was available, as a unique SP ID was recorded on their vaccination card. For the purposes of this study, dogs vaccinated by someone other than MR team were excluded.

#### Other data sources

Poverty data were sourced from two WorldPop raster datasets (http://www.worldpop.org.uk/), where 2010-11 estimates of proportion of people per grid square living in poverty, as defined by $1.25 a day and $2 a day thresholds respectively are available [23]. Land cover data were sourced from the MASDAP Malawi Landcover 2010 Scheme I raster dataset (http://www.masdap.mw/). Land use data was sourced from OpenStreetMap data downloaded on 10th April 2017 (www.openstreetmap.org).

### Data analysis

All data analysis was carried out within the R statistical software environment [24]. Specific packages used are mentioned below.

#### Distance calculation

Each SP location was assigned a unique four digit code which was clearly recorded on every vaccination certificate dispensed from that SP. The owners of any dogs encountered at the door-to-door vaccinations who stated that their dogs had already been vaccinated at SP were required to show their vaccination card. If present, the unique SP ID was recorded in order to be able to link the dog’s home to which SP the dog was vaccinated at. Distance between SP locations and dog’s home was calculated in two ways: a) a straight line distance between the two geographical locations, b) a walking path distance.

For the former we calculated the euclidian distance in km between two polar coordinates. The latter was estimated by using the Google Maps Directions API (https://developers.google.com/maps/documentation/directions/intro) in combination with the *RJSONIO* package [25] to enable us to make multiple google maps enquiries at once. The Directions API was set to provide the shortest walking route between two geographical locations, as well as the distance of that route in km.

In order to investigate whether missingness of vaccination cards was related to how long one had to walk to get home, the distance to nearest SP between those who kept their vaccination and those who lost it was compared using a two sample t-test.

#### Nearest SP to dog home location

For each dog home location recorded at the door-to-door the nearest and second nearest SP was identified using *RANN* package [26]. This enabled us to first estimate the number of dogs taken to their nearest and second nearest SP. Secondly, using the whole dataset we estimated the distance in km to every dog’s nearest SP to be used in the statistical model described below.

#### GIS data extraction

GPS coordinates recorded for each dog identified at door-to-door were used to extract the GIS data for that dog. The package *sp* [27, 28] was used to extract data from shapefiles, while package *raster* [29] was used to extract data from rasters.

#### Multivariable logistic regression model

A multivariable logistic regression model was built using attendance at SP vaccination clinic as the dependent variable. Explanatory variables included distance to nearest SP location in km, housing density and proportion of population counted as poor. As mentioned above, for every dog encountered during the door-to-door campaign, information on whether the dog was previously vaccinated by someone other than MR or whether the dog was vaccinated at a SP was recorded. Dogs recorded to be previously vaccinated were removed from the dataset and the others where categorised as either “vaccinated at SP”, “not vaccinated at SP”, which was used as the model’s outcome variable. Data used for this analysis are shown in S1 Dataset.

The dataset was split into a training dataset (60%), which was used to build the model and a test dataset (40%), which was used to validate the model using that *caret* package [30]. Variable selection was carried using manual forward selection based on lowest AIC. If variables were highly correlated, only one of those variables was included in the final model. SP-level random effects were included to adjust for differences between the SPs. Relevant interactions were also considered. The final model was validated, testing its ability to predict attendance to SP in the test dataset by estimating the area under the curve using package *ROCR* [31].

#### Reasons for not attending vaccination clinics

Information on the reasons for not attending SP clinics, was acquired for any dog found in the D2D campaign, that was not previously vaccinated and did not attend a SP vaccination clinic. A summary of this data is presented. Additionally, using this subset of data, the relationship between people being unaware of the SP vaccination campaign and distance to their nearest SP was explored using a two sample t-test.

## Results

### Demographics

22,924 dogs recorded during the door-to-door campaign were used for this analysis. This excluded 700 dogs that had been vaccinated by someone other than MR earlier that year. 91% included in the analysis were adults and 10% were neutered. 40% of dogs were female out of which 13% were either pregnant or lactating. The vast majority of dogs were owned, out of which 24% were recorded as always roaming, 34% as roaming daily but restricted at some point during the day, less than 1% as roaming weekly and 33% as never roaming.

**Fig 1:**
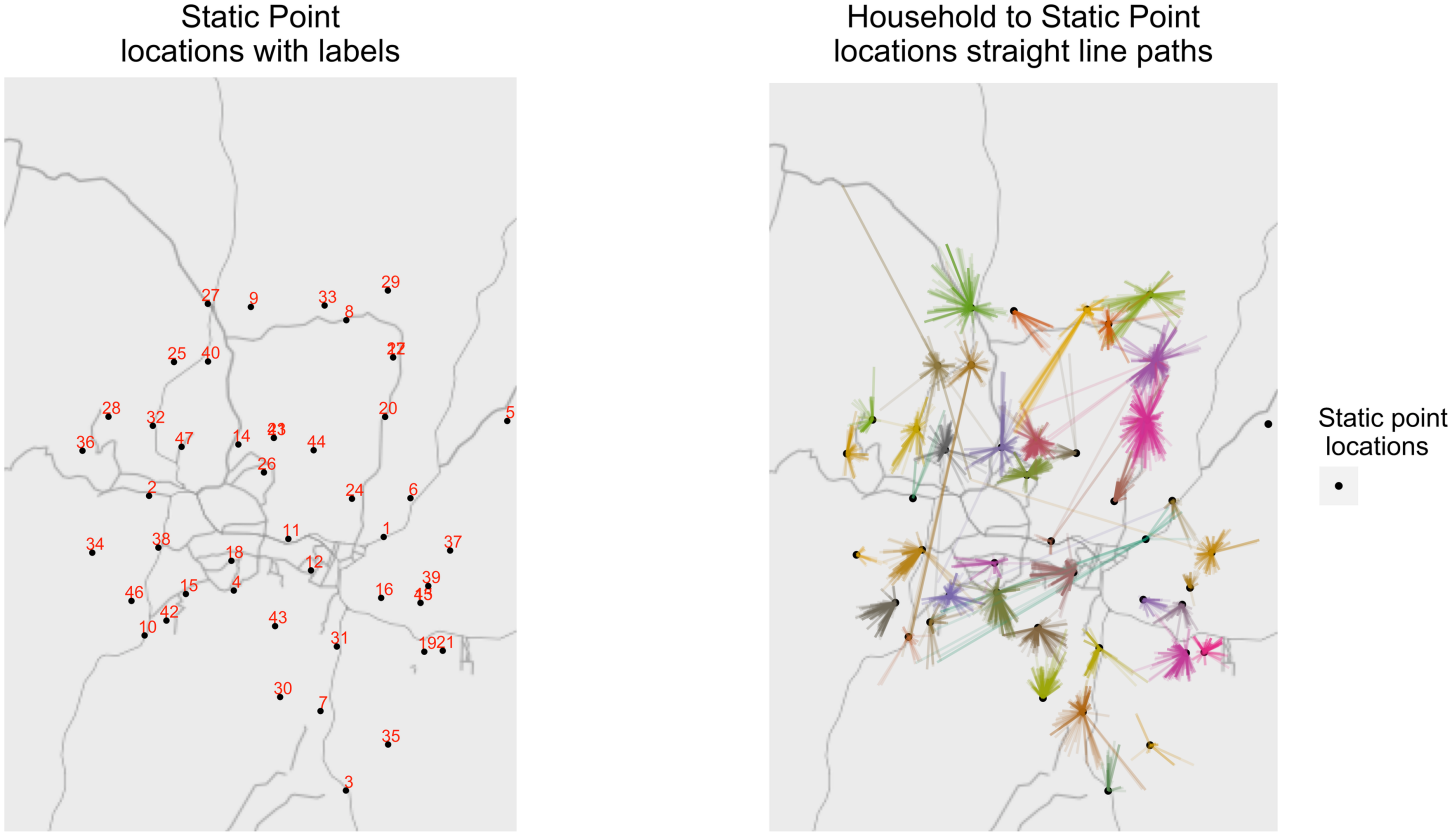
SP locations and recorded straight line paths. Location of SPs used in the study. Examples of the straight line paths for the 6,271 dog owners who presented their SP unique ID number during the door-to-door campaign. The map was plotted using R packages *ggplot2* and *ggmap* [32] using tiles sourced from Stamen Design (using data by OpenStreetMap).

10,476 dogs (0.46) were reported to be vaccinated at SP locations and for 6,271 of those we could retrieve their unique SP location ID. 4,756 (0.76) of those went to their nearest SP location, while 994 (0.16) went to their second nearest SP. Fig 1 shows the location of the SPs used in this campaign, as well as household to SP paths drawn for people who presented their unique SP ID number during the door-to-door campaign. Lastly, there was no statistical evidence of a relationship between distance to the nearest SP and missingness of vaccination card evidence.

### Walking distance to SP

**Fig 2:**
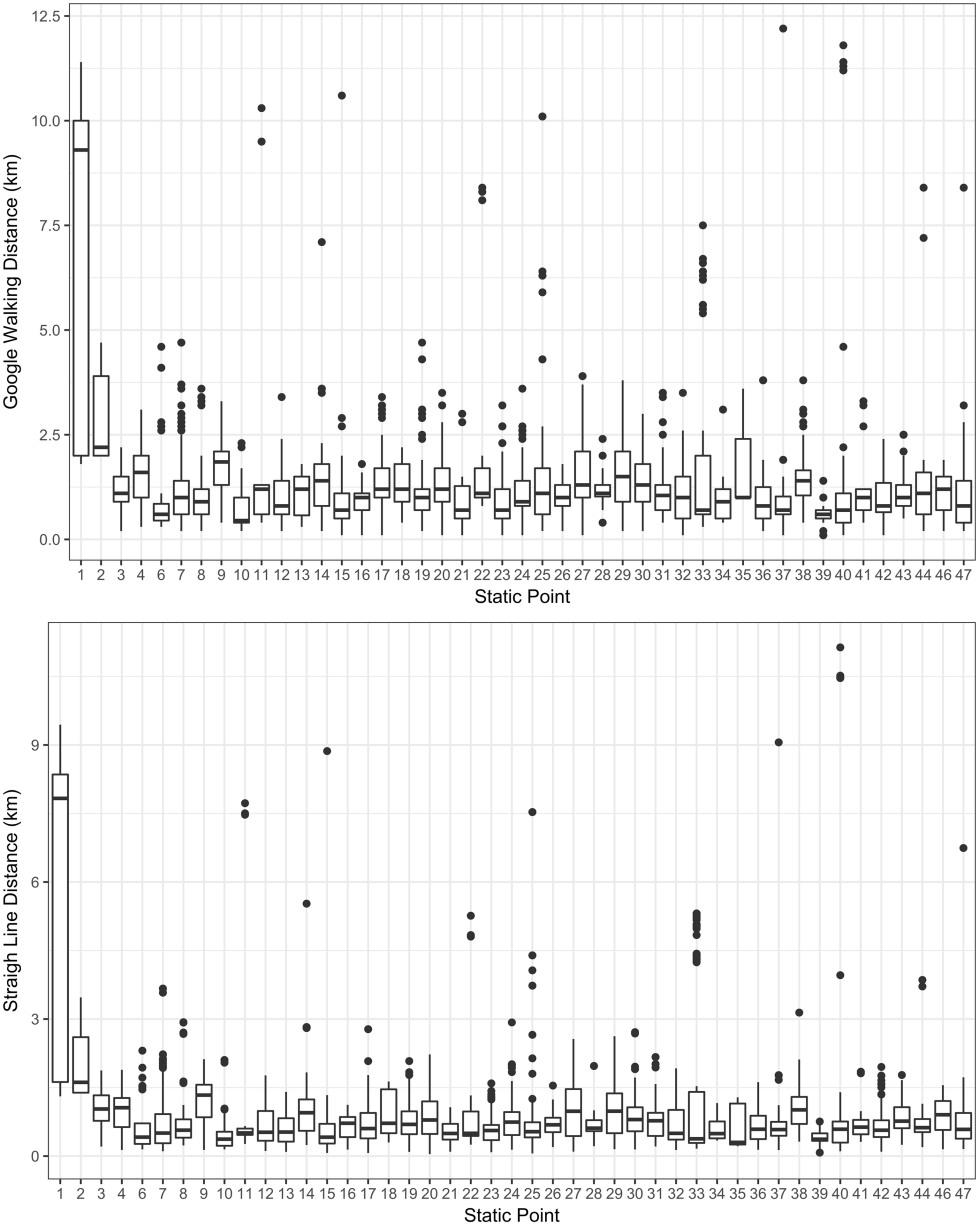
Distance by SP location. This figure shows distance traveled to each of the 47 SP clinics calculated using google maps and straight line distance. Box plots show lower and upper quartiles and the median distance traveled.

Based on the Google Maps route the distance attending dog owners were willing to travel to a SP vaccination clinic was on average 1.22 km with 75% of attending dog owners walking up to 1.5 km to the SP. Similarly, the mean straight line distance was estimated to be 0.812 km with an upper quartile of 1.016 km. S1 Fig demonstrates the difference between the two methods for calculating the distance and demonstrates why distance estimates can vary using the two methods. Fig 2 shows distance traveled to each of the 47 SP clinics, using the two distance estimation methods. It shows that there is great variation in the range of distances SPs manage to attract individuals from. This implies that there might be underlying reasons why some SPs attract individuals from much greater distances than other. In order to ensure that these differences are adjusted for, the addition of SP-level random effects was considered in the regression model.

### Univariable analysis

**Fig 3:**
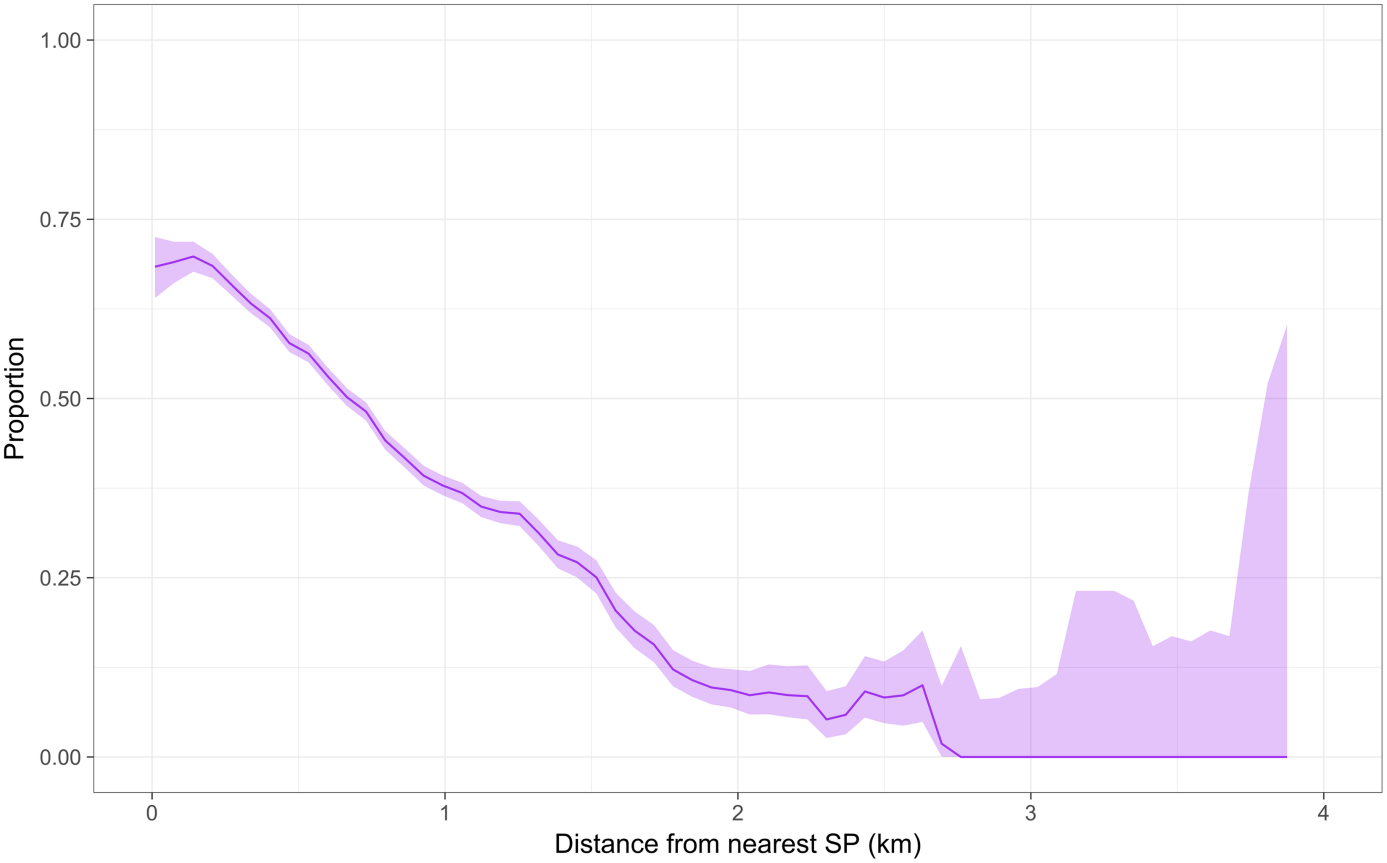
Proportion of attendance to SP by distance to nearest SP. Figure shows that the proportion of attendance to SP decreases as distance to nearest SP increases.

Data used to build a multivariable logistic regression model predicting attendance to a SP included dog related data, household related data extracted from poverty, land use and land type GIS data and straight line distance from nearest SP. A summary of data used as predictor variables is presented in S1 and S2 Tables. Furthermore, Fig 3 shows how the proportion of attendance to SP decreases as distance from nearest SP increases.

Univariable analysis results are shown in Table 1. Land use data were not considered for the model as almost all dogs were located within residential areas. Similarly, ownership status was excluded as very few of the dogs seen were strays (1%). All other variables were considered for the final model.

**Table 1:** Univariable analysis results of the association of each factor with attendance to SP.

Variable	OR	LL	UL	pv
**Distance to SP (km)**				
	0.23	0.21	0.25	<0.001
**Housing Density**				
Low	1			
Medium	2.54	2.37	2.73	<0.001
High	2.86	2.64	3.1	<0.001
**Land Cover**				
Forestland	1			
Grassland	2.05	1.31	3.23	0.002
Cropland	1.34	1.05	1.71	0.018
Settlement	3.84	3.07	4.8	<0.001
**Poverty: proportion of people living in poverty (earning < $1.25/day)**				
q1 (0.034—0.053)	1			
q2 (0.053—0.073)	1.65	1.53	1.77	<0.001
q3 (0.073—0.12)	2.03	1.89	2.19	<0.001
q4 (0.12—0.47)	1.32	1.23	1.43	<0.001
**Sex**				
female	1			
female_preg/lact	0.78	0.69	0.87	<0.001
male	1.13	1.07	1.2	<0.001
unknown	1.45	1.21	1.73	0<0.001
**Age**				
Adult	1			
Puppy	0.3	0.28	0.33	<0.001
**Ownership status**				
Owned	1			
Stray	0.82	0.63	1.06	0.124
**Confinement level**				
always roaming	1			
daily roaming	1.13	1.05	1.21	0.001
weekly roaming	1.23	0.89	1.7	0.213
never roaming	0.8	0.75	0.86	0
unknown	1.89	1.67	2.14	0
**Confinement level**				
Never Roaming	1			
Roaming	1.34	1.27	1.42	<0.001
**Neuter status**				
Entire	1			
Neutered	1.56	1.42	1.72	<0.001
Unknown	3.03	2.69	3.42	<0.001
**Health status**				
Disease present	1			
Healthy	1.7	1.55	1.86	<0.001
Unknown	10.22	8.77	11.9	<0.001

### Multivariable logistic regression model

**Fig 4:**
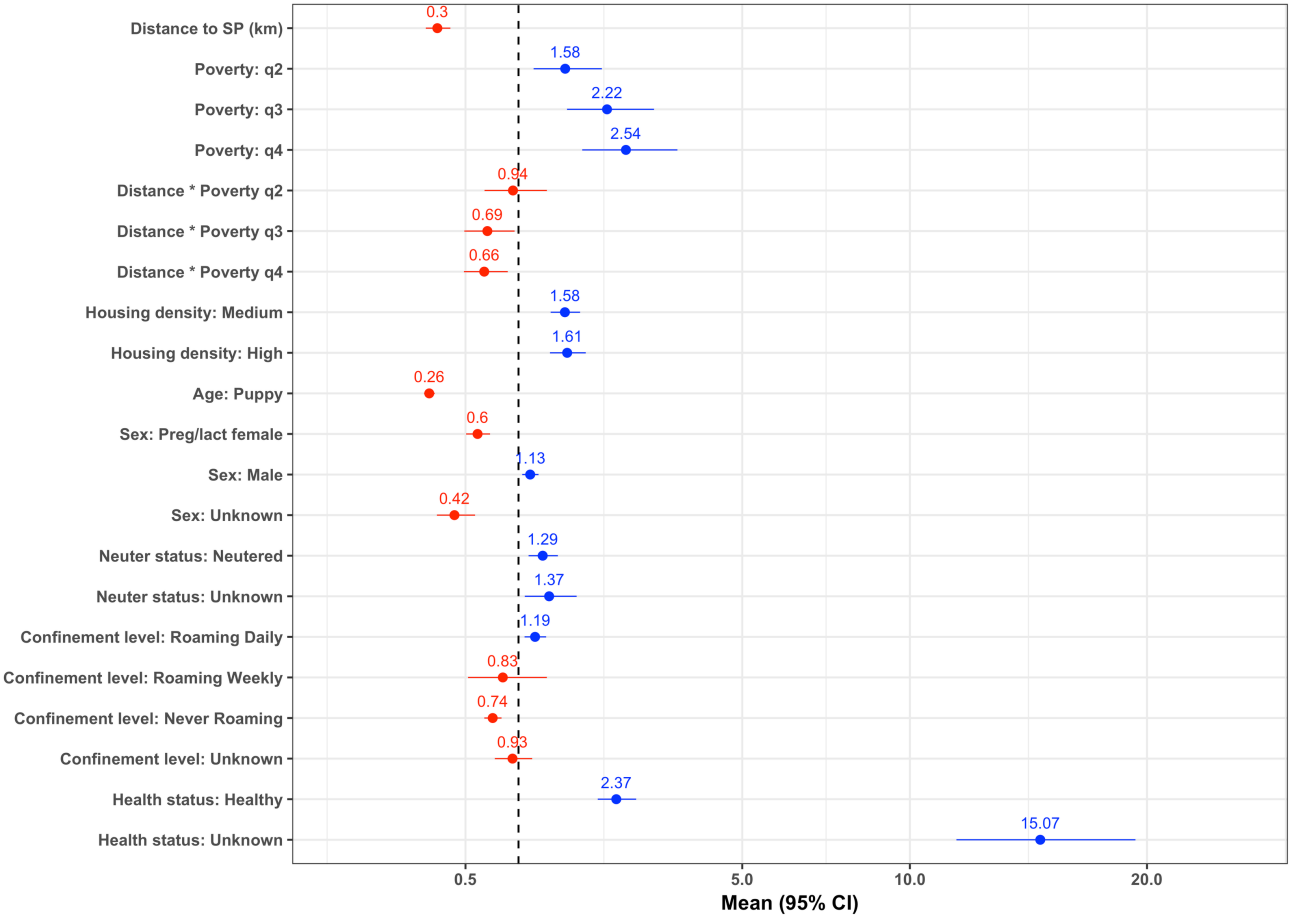
Multivariable logistic regression model predicting attendance to SP. Multivariable logistic regression model predicting attendance to SP using 13,544 observations included in the training dataset.

Fig 4 shows the final multivariable logistic regression model predicting attendance to SP. Numerical results of the regression model can be found in S3 Table. While increasing distance from SP, being a puppy or pregnant/lactating decreased the odds of a dog being taken to a SP for vaccination, high proportions of poor people among a region, as well as living in a high and medium housing density area were positive predictors of attendance to SP. The model also showed that the effect of distance was increased with increasing levels of poverty i.e. there was an increased drop of attendance with distance in poorer people. Regarding dog characteristics being healthy or neutered increased the odds of a dog being taken to SP for vaccination. Lastly, compared to dogs who always roamed, dogs who were reported as never roaming were less likely to be taken to a SP, while dogs who were allowed to roam daily, but restrained for part of the day had increased odds for being taken to a SP for vaccination. The predictive ability of the model was assessed by using the model to predict whether a dog was taken to a SP or not using the test dataset. The AUC was calculated as 0.77, indicating that the model was reasonably good at predicting the outcome.

### Reasons for not attending vaccination clinics

**Fig 5:**
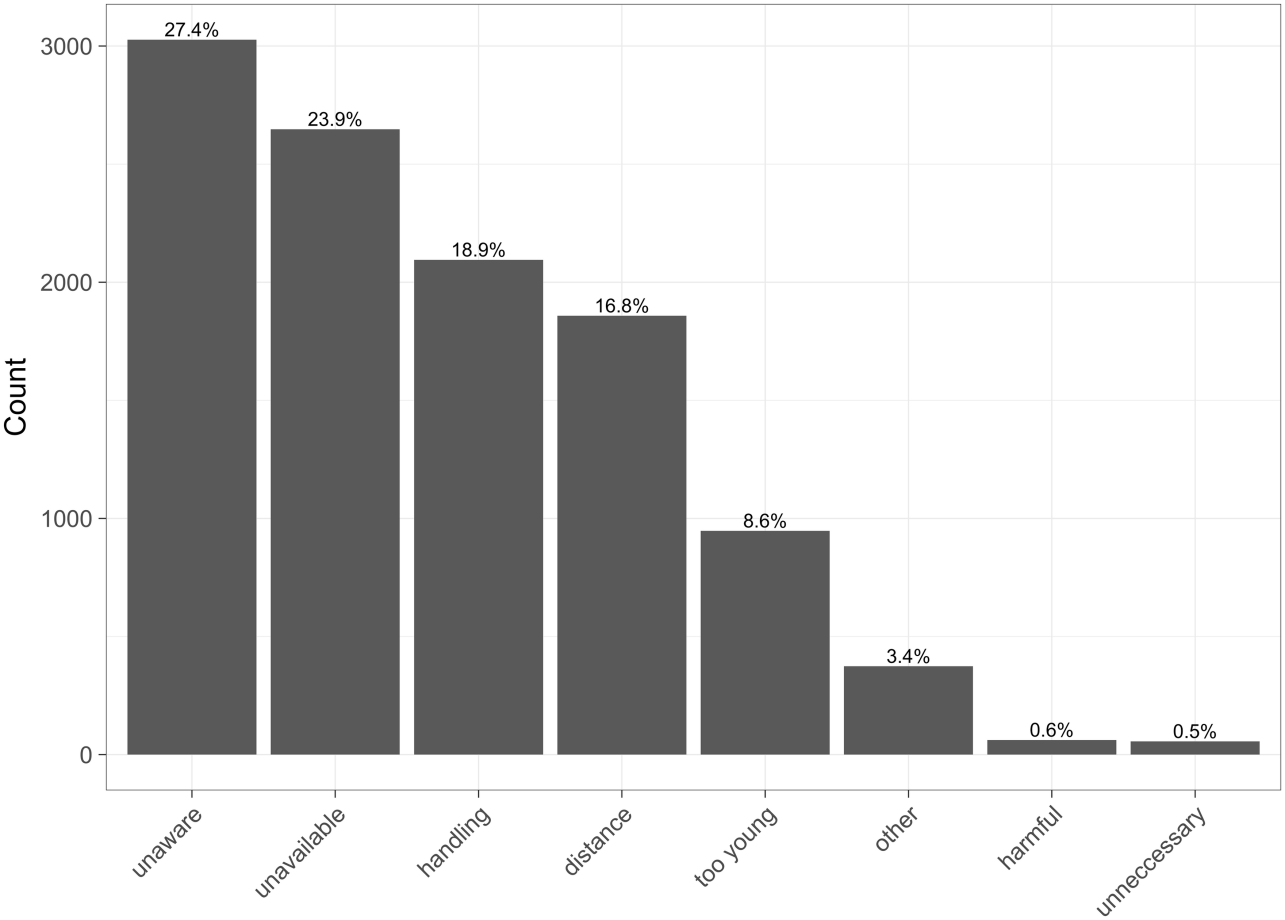
Quoted reasons for not attending SP. During the door-to-door survey owners where asked for the reasons for not attending a SP. This figure presents the main reasons quoted by the dog owners (N = 11,067).

During the door-to-door survey, people who did not attend a SP clinic were asked why. Reasons quoted for not having attended a SP clinic are shown in Fig 5. The most common ones included the owners being unaware, unavailable or unable to handle their dogs, distance and the puppies being too young. This result complements the results of our model by emphasising the importance of distance and the fact that the age of the dog will influence their decision on whether to bring it for vaccination. It also provides further information on other possible reasons why dogs might not be presented at a SP clinic, which our model was unable to take into consideration. These include owner related factors such as lack of awareness, availability and difficulty in handling dogs.

Investigating further into the relationship between people being unaware of the SP vaccination campaign and distance to their nearest SP, it was found that people who said that they did not attend the SP because they were not aware of the campaign were located further to a SP than those who quoted different reasons for not attending (mean unaware = 1.10 km, mean other reason = 0.92 km, p-value = < 0.01).

## Discussion

This paper presents the results of the first large scale study investigating the reasons why attendance at SPs offering free rabies vaccinations for dogs is suboptimal in SSA. We were able to interrogate data from a city-wide vaccination campaign in Blantyre, Malawi using a combination of GIS and household questionnaire type data. We found that distance from household played an important part in SP attendance. Specifically, our regression model showed that for every km closer the odds that the dog was taken to a SP for vaccination were 3.3 times higher. Distance was also one of the main reasons for not attending SPs most commonly quoted by the owners (17%). This finding has been replicated in other studies, which also found distance decay in the use of health services in developing countries [33–37]. Our findings were consistent with smaller scale studies in rural Tanzania, where vaccination coverage decreased as distance from sub-village [18] or household [13] to SP increased.

In order to better inform the planning of future vaccination campaigns we also estimated the distance people were actually willing to walk to a SP, using data from 6,271 dogs for which we could retrieve vaccination cards with SP IDs. Our approach was unique in mapping both straight line distance and actual path based distance. We estimated that people were willing to travel on average 1.22 km to a SP vaccination clinic with 75% of the people walking up to 1.5 km to the SP. Similarly, the mean straight line distance was estimated to be 0.812 km with an upper quartile of 1.016 km. This information is crucial and should be used in planning efficient vaccination campaigns in urban sub Saharan settings in order to improve vaccination coverage using SPs only. In addition, our study highlighted the different uses of straight line distance versus path distance. While straight line distance is very useful when designing mass campaigns as it is much easier to estimate, path distance is more accurate and would be a more valuable tool in estimating for example the cost of travel of each SP attendant. Our study clearly demonstrates that the path distance is on average 50 per cent greater than the straight line distance in this setting.

We also found that socio-economic status influenced attendance to SP vaccinations. Our model shows that dogs from areas with higher proportions of people living in poverty are more likely to be presented for vaccination. Interestingly, the model also shows that the effect of distance described above is increased at increasing levels of poverty. In other words, there is an increased drop of attendance with distance in areas with higher proportions of people living in poverty. This is the first study to report this relationship, which highlights the importance of understanding more about which groups of people might be more inclined to bring their dog to a SP for vaccination. The only other study that has looked at this relationship has found no difference in vaccination coverage between households with high and low socio-economic status in rural Tanzania [13]. The conflicting results might arise due to the fact that our study was carried out in an urban setting. According to our experience in Blantyre, dogs are often brought to SPs by younger members of the family. Middle and high income parents might be less inclined to send their children alone to a vaccination point. Similarly, affluent people may consider their time more costly and be less willing to spend it waiting in queues in order to get their dogs vaccinated.

The signalment of the dogs was also important in influencing likelihood of attending a SP. Our model shows that young dogs, pregnant or lactating females were less likely to be brought to SP vaccination stations. Young age was also reported by 9% of the owners themselves as a reason for not bringing a dog to a SP both in our study and other studies in SSA [16–18]. Puppies less than three months old are often excluded from vaccination campaigns, either due to the misconception that they cannot mount an immune response or because it would require administration of the vaccine off-label [38]. Nevertheless, previous experimental and field studies have shown that puppies can mount a protective immune response as young as 4 weeks old [38–40]. Puppies constitute up to 30% of the dog population in SSA [11] and can therefore play a crucial role in maintaining vaccination coverage beyond the 70% threshold. In fact, WHO guidelines on mass vaccination campaigns advise vaccinating all dogs including those under three months of age [10]. This important issue needs to be addressed through improved advertising and education in order to increase vaccination coverage by ensuring that puppies as well as adult dogs are presented to static vaccination points.

Another interesting finding was the relationship between the reported dog confinement level and SP attendance. We found that compared to dogs who always roamed, dogs reported as never roaming were less likely to be taken to a SP. This might be because people believe that if dogs are not allowed to roam, they are not at risk of rabies. While this might be true for dogs that are kept in a protected area, many dogs will just be kept on a leash or in a garden where other dogs have access to and are therefore at risk of contracting rabies. This is another important issue to be raised during rabies education sessions and vaccination campaign advertisement. In comparison, dogs who were allowed to roam daily, but restrained for part of the day had increased odds of being taken to a SP for vaccination when compared to dogs who roamed all the time. This might reflect the fact that people who interact more with their dogs, are also keen to provide health care or simply be proxy for whether people were able to handle their dogs in order to bring them to the SP.

We also found that a lack of awareness of the vaccination programme was important despite high local profile within the local media, communities and schools. SP vaccination stations were advertised using posters and local radio during the weeks preceding the campaign and announced using a loud speaker in the communities around each station in the days before the actual vaccinating teams arrived at the SPs [20]. Despite these efforts, the most common reason for not attending a SP quoted by the owners (27%) was that they did not know about it. In fact, people further away from SP were less likely to be aware of the vaccination campaign. Promotion of a vaccination campaign is massively important and indeed being unaware was one of the most commonly quoted reasons for failure to attend a vaccination SP in other developing countries including Chad [16, 17], Mali [14], Tanzania [18] and Peru [15]. Timely and accurate provision of information about upcoming SP vaccination stations is likely to increase participation at SPs, and might therefore be cost-effective for future campaigns to invest a greater proportion of resources on campaign advertisement and promotion making sure they cover the area of interest homogeneously.

Another important reason for not bringing a dog to a SP identified by 19% of the owners in this study was difficulty in dog handling. This supports findings of previous studies in developing countries [13–15, 17, 18]. In settings where most dogs are owned for guarding or hunting [11], dogs may be less accustomed to being walked on a leash, making it very difficult for owners to bring them to SP vaccination stations. In order to achieve greater coverage at SP this problem cannot be ignored. Promotional campaigns and rabies education work need to include information on how to safely handle and walk dogs. Such information might need to be provided throughout the year in order for the dogs to be more likely to be able to be handled at vaccination time. Examples of programmes focusing on improving dog handling have been used in several countries in Latin America [15], but have not been previously described in SSA possibly due to economic constraints. With rapidly rising mobile phone ownership in SSA, regional mass SMS delivery through the most popular networks has the potential to greatly increase dissemination of information about time and location of up and coming SPs and therefore possibly increase turn-out.

The present study has several strengths and limitations. Data used for our regression model were sourced from an intensive vaccination campaign which aimed to cover the whole city and is therefore likely to be very representative of the dog population in Blantyre city. This provided us with data about each dog’s signalment as well as GPS locations used to estimate distance to nearest SP and extract GIS data corresponding to each location. This resulted in a detailed dataset and enabled us to extensively explore factors affecting attendance at SP locations. Our model validation showed that our model was reasonably good at predicting the outcome, but there was some unexplained variation. This might have arisen due to the fact that GIS data sources for Malawi are limited and not very detailed or due to information we did not collect such as households who did not respond, number of dogs per households and whether they had equipment to restrain dogs. Similarly, this might be due to information we were unable to include in this kind of model and indeed our household questionnaire showed that two of the most common reasons for not attending SP were being unaware of the campaign and having difficulty in handling which were not included in the initial model. Lastly, we have used google maps to calculate the path distance people were willing to travel to SP stations. This is an innovative way of estimating path distance, which has not been used in rabies relevant studies before, providing a more realistic estimate compared to straight line distance. Nevertheless, it is important to remember that the accuracy of this estimate greatly depends on the accuracy of google maps data in each region and might not be applicable in all areas.

## Conclusion

Overall, this is first large scale study investigating the barriers to obtaining adequate rabies vaccination coverage through SPs in an urban setting in SSA. Our results suggest that future vaccination campaigns should increase efforts on improving positioning of SPs so that they become more accessible. We have also shown that there is a clear need to provide timely and accurate information about upcoming campaigns, emphasing the importance of puppies being vaccinated and identifying ways to improve dog handling. Estimates from our model could be used to estimate the impact on vaccination coverage of adapting several measures such as increasing vaccination points or increasing the proportion of puppies vaccinated, however caution should be exercised due to potential factors not accounted for by the model. In conclusion, this study has provided valuable insight into the barriers to attendance at SPs in urban settings and this should be taken into consideration when designing future mass vaccination programmes using SP vaccination stations in order to allow high vaccination coverage to be achieved without the need for expensive and logistically challenging door-to-door programmes.

## Supporting information

S1 Dataset Dataset. Csv file containing spreadsheet of data used for this analysis. Please note that GPS locations were removed for protection of privacy.
(CSV)

S1 Fig Distance estimation explanation. Figure explaining the two methods used for distance calculation between two locations.
(PDF)

S1 Table Data summary of categorical predictor variables. Cross-tabulations of categorical predictor variables against attendance to SP including missing data (NA) if any.
(PDF)

S2 Table Data summary of continuous predictor variables. Table presents the summary of continuous predictor variables including missing data (NA) if any.
(PDF)

S3 Table Results of the final multivariable logistic regression model predicting attendance to SP. Estimates and 95% confidence intervals of the final multivariable logistic regression model predicting attendance to SP.
(PDF)
