# Supplementary material for: Barriers of attendance to dog rabies static point vaccination clinics in Blantyre, Malawi
Source: PLoS Negl Trop Dis. 2018 Jan 11;12(1):e0006159. doi: 10.1371/journal.pntd.0006159 (PMC5783422; doi:10.1371/journal.pntd.0006159)
Supplement: S1 Fig — Figure explaining the two methods used for distance calculation between two locations. (PDF) [file pntd.0006159.s002.pdf]

Demonstration of methods used to estimate distance between two points

In this study distance between two points was estimated using two methods, namely straight line distance (euclidian) and google maps path distance. Figure S1 describes what is referred to as straight line distance and google maps path distance. A straight line distance is simply the distance when one joins the household location to the SP location (triangle) with a straight line (left). The google maps path to estimate the distance, calculates the km along the fastest walking path google maps come up with (right). The benefit of this is that it calculates a more realistic distance as there might be barriers causing someone to walk round them, hence not walk in a straight line. The disadvantage is that it is as accurate as the detail of the road/walking path data available to google maps. For example very small walking paths or shortcuts are likely to be excluded, especially if crossing property.

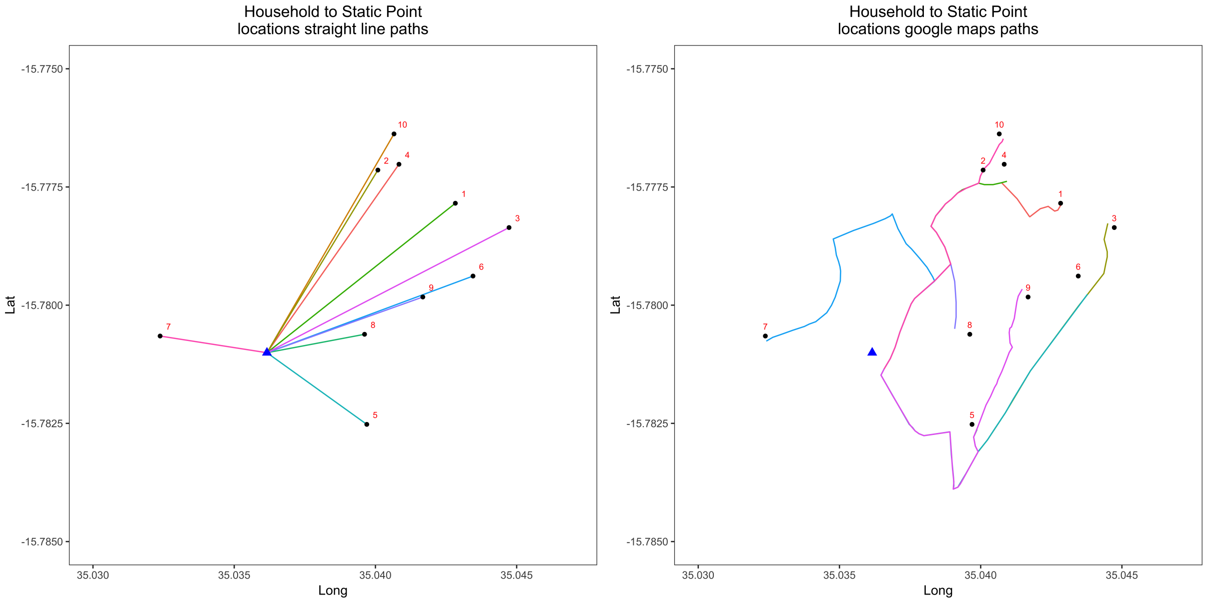

Figure S1: **Distance calculation explanation.**
Figure explaining the two methods used for distance calculation between two locations.
