# Supplementary material for: Barriers of attendance to dog rabies static point vaccination clinics in Blantyre, Malawi
Source: PLoS Negl Trop Dis. 2018 Jan 11;12(1):e0006159. doi: 10.1371/journal.pntd.0006159 (PMC5783422; doi:10.1371/journal.pntd.0006159)
Supplement: S1 Table — Cross-tabulations of categorical predictor variables against attendance to SP including missing data (NA) if any. (PDF) [file pntd.0006159.s003.pdf]

Table S1: Data summary of categorical predictor variables. Cross-tabulations of categorical predictor variables against attendance to SP including missing data (NA) if any.

Variable	Yes	No	Proportion Attended SP
**Housing density**			
Low	1473	3661	28.69%
Medium	6039	5904	50.57%
High	2939	2556	53.48%
NA	25	327	7.1%
**Land cover**			
Forestland	98	397	19.8%
Grassland	37	73	33.64%
Cropland	544	1645	24.85%
Settlement	9797	10333	48.67%
NA	0	0	
**Land use**			
farm	5	57	8.06%
forest	1	6	14.29%
industrial	1	28	3.45%
nature_reserve	2	2	50%
residential	9113	9805	48.17%
scrub	0	2	0%
NA	1354	2548	34.7%
Poverty < $1.25			
q1 (0.034 - 0.053)	2116	3662	36.62%
q2 (0.053 - 0.073)	2782	2926	48.74%
q3 (0.073 - 0.12)	3139	2674	54%
q4 (0.12 - 0.47)	2439	3186	43.36%
NA	0	0	
**Sex**			
female	3651	4617	44.16%
female_preg/lact	542	880	38.12%
male	6006	6709	47.24%
unknown	277	242	53.37%
NA	0	0	
**Age**			
adult	9543	9402	50.37%
puppy	933	3046	23.45%
NA	0	0	
**Ownership status**			
owned	10379	12307	45.75%
stray	97	141	40.76%
NA	0	0	
**Confinement level**			
always roaming	2499	2950	45.86%
daily roaming*	3593	3762	48.85%
weekly roaming	77	74	50.99%
never roaming	3486	5149	40.37%
unknown	821	513	61.54%
NA	0	0	
**Neuter status**			
entire	8525	11197	43.23%
neutered	995	837	54.31%
unknown_neuter	956	414	69.78%
NA	0	0	
**Health status**			
disease	705	1525	31.61%
healthy	8335	10619	43.97%
unknown_health	1436	304	82.53%
NA	0	0	

*Dogs allowed to roam every day, but confined at some point during the day. This was often referring to dogs confined during the day, who were allowed to roam at night.
