# Supplementary material for: Barriers of attendance to dog rabies static point vaccination clinics in Blantyre, Malawi
Source: PLoS Negl Trop Dis. 2018 Jan 11;12(1):e0006159. doi: 10.1371/journal.pntd.0006159 (PMC5783422; doi:10.1371/journal.pntd.0006159)
Supplement: S2 Table — Table presents the summary of continuous predictor variables including missing data (NA) if any. (PDF) [file pntd.0006159.s004.pdf]

Table S2: **Data summary of continuous predictor variables.** Table presents the summary of continuous predictor variables including missing data (NA) if any.

	**Min**	**Median**	**Mean**	**Max**	**NA**
**Distance to SP (km)**	0.01	0.75	0.83	6.50	-
**Poverty < $1.25**	0.03	0.07	0.10	0.47	-
**Poverty < $2.00**	0.13	0.25	0.30	0.76	-
