# Supplementary material for: Barriers of attendance to dog rabies static point vaccination clinics in Blantyre, Malawi
Source: PLoS Negl Trop Dis. 2018 Jan 11;12(1):e0006159. doi: 10.1371/journal.pntd.0006159 (PMC5783422; doi:10.1371/journal.pntd.0006159)
Supplement: S3 Table — Estimates and 95% confidence intervals of the final multivariable logistic regression model predicting attendance to SP. (PDF) [file pntd.0006159.s005.pdf]

Table S3: **Results of the final multivariable logistic regression model predicting attendance to SP.** Estimates and 95 % confidence intervals of the final multivariable logistic regression model predicting attendance to SP.

**Variable**	**Odds Ratio**	**Lower 95% CI**	**Upper 95% CI**	**p value**
**Distance to SP (km)**	0.30	0.24	0.39	0.00
**Poverty < $1.25***				
Poverty: q1	Reference			
Poverty: q2	1.58	1.17	2.13	0.00
Poverty: q3	2.22	1.61	3.06	0.00
Poverty: q3	2.22	1.61	3.06	0.00
**Distance * Poverty**				
Distance * Poverty q1	Reference			
Distance * Poverty q2	0.94	0.66	1.34	0.73
Distance * Poverty q3	0.69	0.49	0.96	0.03
Distance * Poverty q4	0.66	0.49	0.89	0.01
**Housing density**				
Low	Reference			
Medium	1.58	1.39	1.80	0.00
High	1.61	1.38	1.88	0.00
**Age**				
Adult	Reference			
Age: Puppy	0.26	0.23	0.29	0.00
**Sex**				
Female	Reference			
Sex: preg/lact female	0.60	0.50	0.71	0.00
Sex: male	1.13	1.04	1.23	0.00
Sex: unknown	0.42	0.30	0.58	0.00
**Neuter status**				
Entire	Reference			
Neutered	1.29	1.11	1.48	0.00
Unknown	1.37	1.07	1.75	0.01
**Confinement level**				
Always Roaming	Reference			
Roaming Daily	1.19	1.07	1.33	0.00
Roaming Weekly	0.83	0.52	1.34	0.45
Never Roaming	0.74	0.66	0.82	0.00
Unknown	0.93	0.76	1.16	0.54
**Health status**				
Disease	Reference			
Healthy	2.37	2.07	2.72	0.00
Unknown	15.07	11.69	19.43	0.00

*Poverty: proportion of people living in poverty per grid square (earning < $1.25/day)
